# Water-Proof Shoes

**Published:** 1866-12

**Authors:** 


THE UNDERSTANDINGS

of the physical man has a great deal to do with health and life. Many a man and woman owe an untimely death to damp feet in winter-time. This is very generally admitted, and many methods have been proposed to prevent it. In wet weather, or when the snow is melting, the India rubber shoe is the most perfect article offered ; some prejudice has been excited against them, more than anything else from the unwise use of them. They may be hurtful to some, but it does not follow that they are generally so.

No one can be comfortable with cold, damp feet, and the very instant it is noticed, the person should begin to walk, or remove both stockings and hold the bare feet to the fire until they are perfectly dried and feel comfortably warm. India rubber overshoes should be worn only when the person is walking ; as soon as the walk is ended they should be removed. They certainly ought not to remain on the feet ten minutes if the person is standing still in the house after a walk. If a person is in and out many times in a day, it is better to have a pair of old shoes covered with good India Rubber to remain on permanently, to be slipped on and off together ; another pair of shoes and slippers, to be worn in their stead while indoors ; but unless this other pair is always kept warm when not in use, the removal of a shoe and slipping on a cold one, will give a bad cold or a troublesome rheumatic affection in a very short time. The great mass of business men cannot make these changes, and to them the old fashioned soled shoe or boot is best.

Various expedients have been devised to keep the dampness from the soles of the feet. Some advise that a piece of sail cloth or other woven material, should be cut in the shape of the sole, dipped in melted pitch or tar, and when cooled, placed between the layers of the shoe's sole and well sewed. If this is carefully done it is simply impossible for any dampness to penetrate to the soles of the feet by simply walking on damp ground; but in walking in wet grass or the slosh of snow deep enough to reach the upper leather, this device is no protection.

Another means of rendering soles of shoes impervious to dampness, and to prevent their squeaking, is to set them in melted tallow deep enough to merely cover the soles, and let them remain a week; if it is in a mixture of equal parts of beeswax and tallow it is still the better.

A gentleman avers that from six years of experience and trial, the soles of shoes are not only made water-proof but will last three times as long if a coat of gum copal varnish is applied to the soles and repeated as it dries, until the pores of the leather are filled, and the surface shines like polished mahogany.

The soles of shoes may be made impervious to water by rubbing the following mixture into the leather, until it is thoroughly saturated—One pint of boiled linseed oil; half a pound of mutton suet; six ounces of pure beeswax; four ounces of rosin. Melt these over a slow fire, stirring well, and when the shoes are new, warm them and the mixture also, and use.

Or put a pound each of rosin and tallow in a pot on the fire and when melted and mixed, apply while hot, with a painter's brush, to both soles and upper leather. If it is desired that the boots should take a polish immediately, dissolve an ounce of beeswax in a teaspoonful each of turpentine and lamp black a day or two after the boots have been treated with the rosin and tallow, rub over them this wax and turpentine, away from the fire. Thus the exterior will have a coat of wax alone, and will have a bright polish. Tallow and grease become rancid and rot the stitching, and the leather also; while the rosin mixture preserves both.

One pint of linseed oil, a quarter of a pint of turpentine or camphor, a quarter of a pound of beeswax and a quarter of a pound of Burgundy pitch. Melt together with a gentle heat; warm it when it is to be used, and rub it into the leather before the fire, or in the sun.

Or, melt together beeswax and mutton suet, half and half, and rub it in where the stitches are.

Gutta Percha soles are preferred by some. They may be attached thus: dry the old sole, roughen it well with a rasp, and rub on with the finger a thin, warm solution of gutta percha; dry it, hold it to the fire, and then rub on a coat of a thicker solution. Take the gutta percha sole, soften it in hot water, wipe it, and hold both sole and shoe to the fire until warm; lay the sole on gradually, beginning at the toe. In half an hour, pare it neatly with a knife.

But it must be remembered that if you make the upper leather of a shoe water-tight, it is rendered measureably air-tight, and this occasions dampness on the inside, creating ill odors and coldness, while any kind of oily substance must not only rot the material but cause a noisome smell.

To those who are forehanded, and have leisure, it is advised to purchase the shoes to be worn in winter six months before hand, and wear them a little at a time in warm weather; thus they become hardened before winter sets in, and this hardening increases their durability. But before they are once worn in the wet, the soles should be held to the fire until they are well warmed; then warm a little tar in a tin cup, and apply it with a swab to the bottom of the shoe, but not hot enough to burn the leather, then let it be well dried in before the fire. This will never work out while warming the feet; but this tar should be applied the first of each month until May, if the boots are worn much in the wet. This tar penetrates the sole to the eighth of an inch, and renders it almost as hard as horn. Grease of any kind will soften the leather and make it porous. Without this tar application, the first wetting of the soles will contract them and making them fit not so well, sometimes making them too small altogether.

If shoes are heated before the fire, they get hard and wear out very much sooner than if allowed to dry gradually in the upper part of the kitchen or family room, farthest from the fire, or on a shelf, or hung on a nail.

